# Potential Adiponectin Receptor Response Modifier Therapeutics

**DOI:** 10.3389/fendo.2019.00539

**Published:** 2019-08-13

**Authors:** Laszlo Otvos

**Affiliations:** ^1^OLPE LLC, Audubon, PA, United States; ^2^Allysta Pharmaceuticals, San Mateo, CA, United States; ^3^Institute of Medical Microbiology, Semmelweis University, Budapest, Hungary

**Keywords:** active site, agonist, antagonist, fibrosis, insulin sensitivity, nanomolar activity, oral efficacy, signaling

## Abstract

Many human diseases may benefit from adiponectin replacement therapy, but due to pharmacological disadvantages of the intact protein, druggable options focus on peptidic, and small molecule agonists of the adiponectin receptor. Peptide-based adiponectin replacement drug leads are derived from, or resemble, the active site of globular adiponectin. ADP355, the first-in-class such peptide, exhibits low nanomolar cellular activities, and clinically acceptable efficacies in a series of fibrotic and inflammation-derived diseases. The advantage of small molecule therapies, spearheaded by AdipoRon, is oral availability and extension of utility to a series of metabolic conditions. It is exactly the difficulties in the reliability and readout of the *in vitro* measures and the wealth of *in vivo* models that make comparison of the various drug classes complicated, if not impossible. While only a fewer number of maladies could take advantage of adiponectin receptor antagonists, the limited number of these available can be very useful tools in target validation studies. Alternative approaches to direct adiponectin signaling control use upstream adiponectin production inducing therapies but currently these offer relatively limited success compared to direct receptor agonists.

## The Need For Peptide- and Small Molecule-Based Adiponectin Receptor Response Modifiers

**Figure 1:**
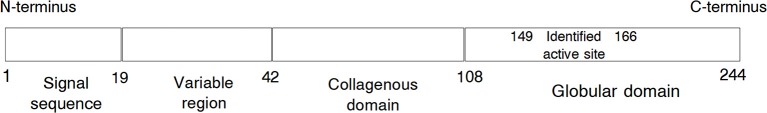
Domain structure of monomeric human adiponectin protein. While multimerization takes place at the N-terminal regions, the identified active site is located at the C-terminal globular fragment.

Adiponectin, a 244 amino acid cytokine, normally produced by the fat tissue is known to exert beneficial effects in a series of human conditions, including insulin resistance, cardiovascular disease, inflammatory conditions and cancer (1–3). The human adiponectin protein is comprised of an N-terminal signal sequence, a variable region, a collagenous domain, and a C-terminal globular domain [(4); Figure 1]. While monomeric forms are infrequently observed, adiponectin protein mainly circulates in trimeric, hexameric and higher order complexes (5). The two adiponectin receptors that are responsible for downstream signaling, AdipoR1 and AdipoR2, are seven-transmembrane domain proteins containing an intracellular domain at the N-terminus and a C-terminal extracellular domain (6, 7).

Due to the generally beneficial effects of the adipokine, a series of human conditions could use adiponectin replacement therapy (8). Prior investigations of adiponectin suggest multiple targets for clinical intervention. Decreased circulating adiponectin concentrations can increase the risk developing type 2 diabetes, metabolic syndrome, atherosclerosis, and cardiovascular disease in obese patients and need to be restored to normal levels (9, 10). Hypoadiponectinemia has also been associated with increased risks of several cancers and correlated with poor long-term prognosis (11). Circulating adiponectin levels are inversely related to inflammation indicating potential therapeutic utility in various conditions characterized with inflammatory processes (12). Adiponectin has demonstratable an anti-fibrotic action in the liver and it was suggested that adiponectin may be developed as a new therapeutic candidate for the treatment of liver fibrosis (13). Lastly, adiponectin impacts important brain functions including energy homeostasis, hippocampal neurogenesis and synaptic plasticity (14). Overall, multiple studies indicate indicate the potential value of adiponectin replacement therapy in the pathohysiology of neuroprotective, antiatherogenic, and antidepressant diseases.

However, there are two salient challenges in converting adiponectin protein or slightly modified versions into a viable drug candidate. Firstly, the heterogeneity of the expressed protein structures (15) prevent highly reproducible results *in vitro* and *in vivo* (16). Secondly, the extreme insolubility of the C-terminal domain and larger peptide fragments thereof (17) prevent druggable protein-based lead generation. Full-length adiponectins produced by mammalian cells, as opposed to bacterially expressed versions, are fully active insulin sensitizers due to post-translational hydroxylation and subsequent glycosylation of lysine residues at the central collagenous domain (18). Adiponectin-based therapeutic modulators are consequently currently unavailable. Luckily, peptide- and small molecule-based adiponectin receptor response modifiers can fill the denoted gap toward clinical development of adiponectin replacement therapies.

## Difficulties in Screening

Comparison of effectors in adiponectin replacement therapies further complicated is by (a) a litany of adopted screening assays (i.e., specific *in vitro* efficacies, various signaling effects, downstream activities) various research groups employ in the enormous number of related reports (19), and (b) the inconsistencies in any given assay looking for downstream effects. For example, cell-type dependence of AMPK activity (perhaps the most frequently used assay for testing adiponectin receptor activation) during adiponectin signaling is reported together with differences in the cellular effects of various adiponectin protein preparations (20). In general, the truthful evaluation of the assay readouts is not without controversy. Many tissues produce adiponectin and locally produced adiponectin can interfere with autocrine or paracrine activities that will result in either false positive or false negative screening assays (21). The proposed alterations in signaling due to differing activation of the two slightly different receptor types (22) interferes with the evaluation of potential adiponectin receptor response modifiers. Age-dependent alterations in adiponectin activity (23) and the involvement of accessory molecules (24) add yet additional aspects to screening and diagnostic difficulties.

Agents with multiple effects also inducing endogenous adiponectin production can exert effects opposite to those predicted, as was shown on acetaldehyde during fibrogenesis in hepatic stellate cells (25). Moreover, adiponectin functions in mouse primary hepatocytes appear to be independent of signaling events observed *in vivo* (26). Furthermore, the extent of adiponectin-induced smooth muscle cell proliferation depends upon proteolytic processing into shorter forms (27).

This review intends to critically evaluate the various adiponectin-replacement therapy options. To make the results more comparable, when EC_50_ or IC_50_ figures were not directly provided in the publications, this author calculated them from the available figures or tables. However, it is beyond the scope of this review to validate the *in vitro* assays and the actual data. The interpretation of the results in the individual reports are taken here *prima facie*. Even so, once preclinical development is underway unforeseen opportunities may arise. The journey of peptidomimetic ADP355 (*vide supra*) from a potential systemic cancer therapeutic to impending clinical trials as eye drops against dry eye disease (28) provides confidence that systematic research on adiponectin functions and potential adiponectin receptor response modifiers may lead to novel therapeutics.

## The Active Site of Adiponectin and First-in-Class Adipor Agonist Peptidomimetics

The first step for designing peptidic agonists of adiponectin receptors was the identification of the active site within the native protein (29). Multiple accounts suggest that the C-terminal globular domain (gAd) carries the active site because this domain exhibits powerful metabolic effects in various tissues (30, 31). Overlapping 10-mers identify an extended stretch of amino acid residues (149–166) that inhibit cancer cell proliferation with the central Ile-Pro-Gly-Leu-Tyr-Tyr-Phe-Ala octapeptide representing the minimally active site [(29); Table 1].

**Table 1:** Sequences of peptidic adiponectin receptor response modifiers.

**Peptide**	**Sequence**
**AGONISTS**	
Globular adiponectin active site	His-Cys-Asn-Ile-Pro-Gly-Leu-Tyr-Tyr-Phe-Ala-Tyr-His-Ile
ADP355	*Asn*-Ile-Pro-Nva-Leu-Tyr-*Ser*-Phe-Ala-*Ser*
ADP399	*Asn*-Ile-Pro-Nva-Leu-Tyr-*Ser*-Phe-Ala-*Ser*-His-Pro-Dab- *Asn*-Ile-Pro-Nva-Leu-Tyr-*Ser*-Phe-Ala-*Ser*-His-Pro
Pep70	Pro-Gly-Leu-Tyr-Tyr-Phe-Asp
BHD1028	Tyr-Tyr-Phe-Ala-Tyr-His-Pro-Asn-Ile-Pro-Gly- Leu-Tyr-Tyr-Phe
**ANTAGONIST**	
ADP400	Chex- Gly-Leu-Tyr-*Ser*-Phe-Ala-*Ser*

*Dab is 2,4-diamino butyric acid, L-Nva is norvaline, Chex is 1-amino-cyclohexyl carboxylic acid. D-amino acids are printed in italics. All designer peptides have free-amino termini and are C-terminal amides*.

A targeted peptide-type library containing non-natural residues to improve metabolic stability and solubility yielded peptidomimetic ADP355, H-DAsn-Ile-Pro-Nva-Leu-Tyr-DSer-Phe-Ala-DSer-NH_2_ (Table 1), that at 100 nM concentration inhibits cancer cell growth better than gAd at 50 ng/mL (~3.5 nM). In agreement with most full-sized adiponectin-related studies, ADP355 increases AMPK and STAT3 phosphorylation in MCF-7 breast cancer cells and inhibits ERK1/2 phosphorylation in MDA-MB-231 triple negative breast cancer cells. A 13-mer peptide derived from the collagen domain of adiponectin also activates AMPK and improves glucose and fatty acid metabolisms but with an EC_50_ of 4 mM on enhancing glucose uptake (32) it cannot be considered a true AdipoR activator (please note the discrepancy between X-axis labeling in μg/mL and actual data in mg/mL).

ADP355 exhibits adiponectin-like activities in several *in vitro* and *in vivo* assays. The peptide inhibits renal fibroblast differentiation with an IC_50_ of ~50 nM concentration (33). In agreement with the reversal of renal fibrosis, ADP355 inhibits fibroblast migration and attenuates fibrotic responses in three-dimensional human skin models together with attenuating constitutive fibrotic gene expression in unstimulated systemic sclerosis fibroblasts (34). Both adiponectin protein and ADP355 suppress phosphorylation of focal adhesion kinase (FAK) in hepatic stellate cells (HSC) assessed by immunofluorescent imaging and quantitation. Because HSCs are precursors for hepatic fibrosis, ADP355 might be an effective therapy option for liver fibrosis (35). In a follow-up study, ADP355 was conjugated to gold nanoparticles and injected intraperitoneally at 1 mg/kg dose (36). Liver fibrosis markers, including serum aspartate aminotransferase, alanine aminotransferase and hydroxyproline are attenuated, suggesting that ADP355 is a potent anti-fibrotic agent and potentially an effective intervention against liver fibrosis. In support, while chronic CCl_4_-treatment induces liver fibrosis, ADP355 treatment significantly reverses this process. Key markers for fibrogenesis (α-smooth muscle actin, α-SMA, transforming growth factor-β1, TGF-β1, connective tissue growth factor, and the tissue inhibitor of metalloproteinase I, TIMP1) are markedly attenuated (36). ADP355 also activates proprotein convertase subtilisin kexin type 9 (PCSK9) and peroxisome proliferator-activated receptor γ (PPARγ) expression at 50 nM (37).

When administered intraperitoneally at 1 mg/kg/day for 14 days to mice with protease inhibitor-induced lipodystrophy, peptide ADP355 restores the subcutaneous adipose tissue and reverses hyperinsulinemia, hypertriglyceridemia, and hypoadiponectinemia (38). In addition, ADP355 prevents protease inhibitor-induced cognitive impairment and brain injury in mice. ADP355 activates hepatic LDLR expression and ameliorates lipid metabolism in both wild type and apoE^−/−^ mice and inhibits atherosclerosis in apoE^−/−^ mice at 1 mg/kg/day administered for 12 weeks (37). Upon intraperitoneal injection into *scid* mice xenotransplanted with MCF-7 breast cancer tumor, ADP355 therapy at a 1 mg/kg/day dose decreases tumor size by 31% compared to untreated animals (29).

In a bleomycin-induced mouse skin fibrosis model, ADP355 treatment mitigates the increase in dermal thickness, collagen accumulation, fibrotic gene expression, and dermal white adipose tissue attrition at both 0.2 and 1 mg/kg/day intraperitoneal doses (34). Liver lysates of mice with liver fibrosis and treated with ADP355 display typical adiponectin-mediated signaling changes such as increased phosphorylation of both endothelial nitric oxide synthase (eNOS) and AMPK as well as decreased phosphorylation of AKT (36). The ADP355 dose in this instance was 0.5 mg/kg intraperitoneally every other day for 14 days. Finally, ADP355 mimics adiponectin action in thioacetamide-induced liver injury (39). ADP355 treatment increases liver glycogen, decreases serum alanine transaminase and alkaline phosphatase activity, and promotes body weight gain, hyper-proliferation and hypo-apoptosis. In addition, 1 mg/kg intraperitoneal ADP355 administration suppresses activation of HSC and liver macrophages. The signaling events are inactivation of TGF-β1/SMAD2 and the promotion of AMPK and STAT3 phosphorylation.

Further optimization attempts of ADP355 failed to yield derivatives with notably improved cellular activities (33, 40). Neither chain extension to the termini, nor reinstallation of native residues, even cyclization of the potent dimer, lead to enhanced activity profile. Incorporation of Pro and Hyp residues changes the conformation to a structure that increasingly resembles to the active native β-sheet. However, the 10-fold improved activity against cancer cell proliferation did not carry over to any enhanced activity in the ophthalmic applications *in vivo*. Switching the ADP355 drug lead that possesses remarkable low dose *in vivo* efficacy and excellent toxicity profiles in various animal models is therefore not warranted.

## Additional Peptidic Adipor Agonists

The C-terminal decapeptide fragment of the active site, termed peptide 27, exhibits *in vitro* and *in vivo* advantages very similar to those elicited by ADP355 in the skin fibrosis model (34). Peptide 27 treatment is associated with increased AMPK phosphorylation in skeletal muscle. Based on the crystal structure of AdipoR1, peptide agonists were designed using protein-peptide docking simulations (41). The sequence of the best hit, BHD-1028 is remarkably similar to a dimeric peptide 27 segment (Table 1). BHD-1028 induces AMPK phosphorylation in differentiated C2C12 myotubes at as low as 800 nM concentration. Dimerization appears to improve the activity of the gAd active site, as a C-terminally tethered version of ADP355 (termed ADP399, Table 1) exhibits a 10-fold decrease of K562 chronic myeloid leukemia cell proliferation rate compared to monomeric ADP355 (33), although this improvement is not obvious in animal models.

Another support for the identification of the correct active site comes from yet another screening of a virtual library against the three-dimensional structure of AdipoR1 (42). The best hit, a 7-mer peptide called Pep70 is strikingly similar to the minimal active site sequence (Table 1). Pep70 inhibits HSC-T6 cell proliferation with an IC_50_ of 5 μM, that is quite similar to ADP355 in this particular measure, casting some doubts to the validity of the biological assay selection.

## First Generation of Small Molecule Adiponectin Receptor Agonists

Not much after the discovery of peptidomimetic ADP355, an extensive study used ADP355 as a reference compound to identify non-peptidic AdipoR agonists from a 10,000-member natural product library. Not surprisingly, even the best hit arctin retains only 3.5 μM activity (43). Additional identified AdipoR1 agonists include arctigenin and gramine. *En route* to pulling out these compounds from the mixture, fluorescence polarization quantified the binding constants of ADP355 to AdipoR forms. Depending upon the location of the fluorescein label and the receptor subtype, the measured K_d_ values range from 800 nM to 1.7 μM. In our experience, the presence of a bulky label decreases the affinity of peptide ligands to their binding partners by 50 to 100-fold compared to naked peptides (44). Thus, these binding assays place the affinity of ADP355 to AdipoR somewhere at the low nM level, in agreement with the results of cellular adiponectin replacement experiments. It needs to be added that according to siRNA inhibition assays, ADP355 activates AdipoR1 stronger than it does AdipoR2 (29), a finding of some difference with the presented fluorescence polarization data.

Parallel to the initial hits, screening of another small molecule library identified an orally available AdipoR agonist called AdipoRon (45). AdipoRon is currently the most extensively studied non-peptidic adiponectin replacement therapy drug candidate.

## Wide Spectrum of *in vitro* and *in vivo* Activities of Adiporon

**Figure 2:**
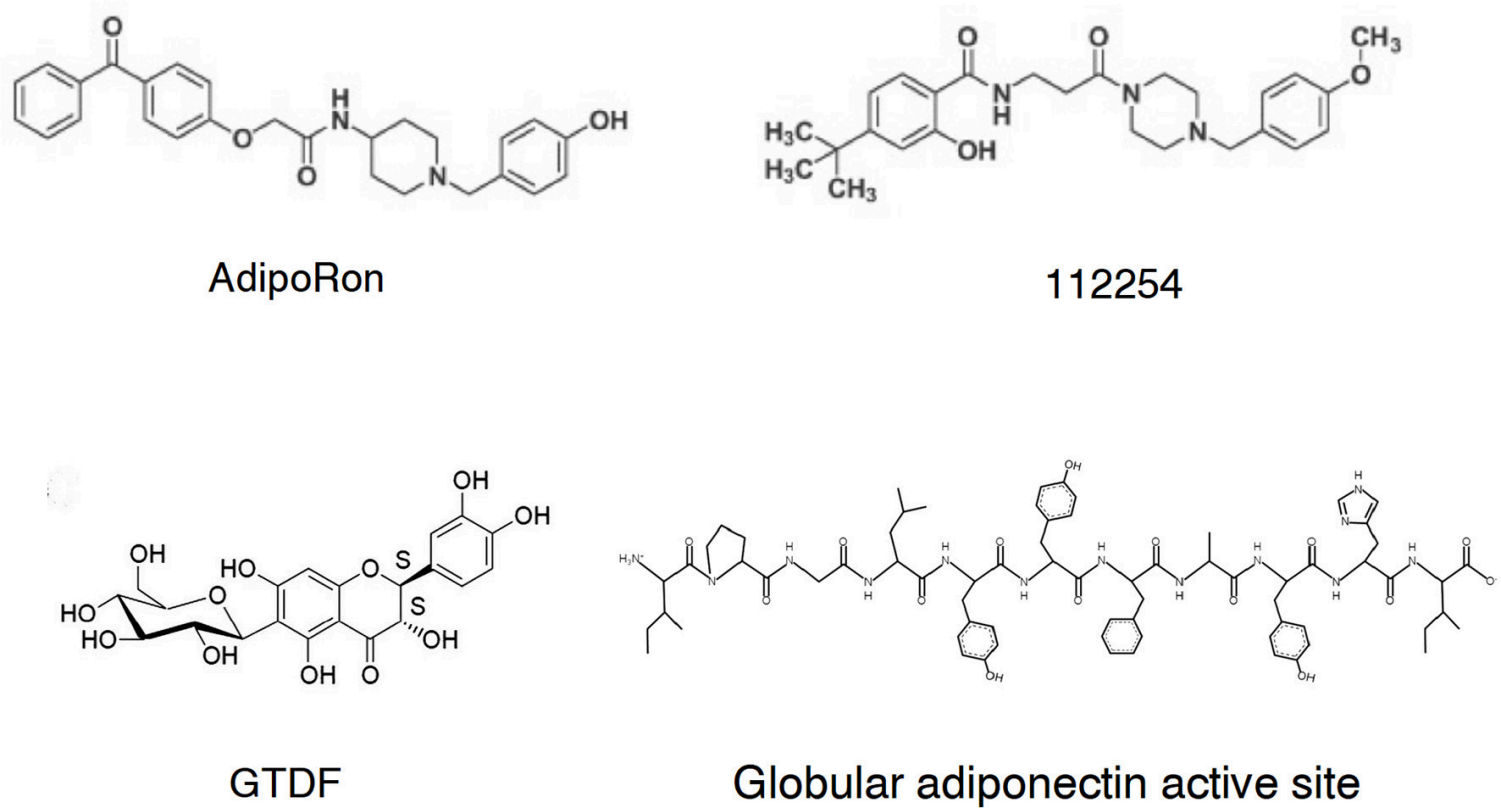
Chemical structures of the globular adiponectin active site and selected small molecule adiponectin receptor agonists.

AdipoRon (Figure 1) was selected from a chemical library at the Open Innovation Center for Drug Discovery in The University of Tokyo (no 108049) based on activation of AMPK and target validation with AdipoR siRNA inhibition in C2C12 myotubes (45). Another hit, no 112254 (Figure 2) has similar *in vitro* activity data, albeit structurally the two compounds are quite different except the p-cresol-piperidine fragment. The binding constants of AdipoRon to AdipoR1 and AdipoR2 are 1.8 and 3.1 μM. The EC_50_ value for AMPK activation is ~10 μM (45) in agreement with the receptor binding data. Similar (5–10 μM) *in vitro* activities were observed in C3H10T1/2 cells, where addition of AdipoRon increases phosphorylated AMPK and ACC levels, the latter being a downstream target of AMPK, and decreases the expression of adipogenic transcription factors C/EBPβ, C/EBPα, and PPARγ (46). Ensuing studies fully support AdipoRon as an adiponectin replacement therapy effector, although current pharmaceutical development go-nogo criteria are not in favor of pursuing a candidate with such a low cellular activity.

In the original report, orally administered AdipoRon at 50 mg/kg to genetically obese db/db mice (lacking the leptin receptor) significantly reduces plasma glucose levels similarly in duration and extent to adiponectin protein injection. At the end of the 2-week treatment while the mice received normal diet, glucose intolerance, insulin resistance and dyslipidaemia are markedly ameliorated without affecting body weight, food intake, liver weight, and white adipose tissue weight. When the diet is switched to high fat content, AdipoRon rescues obesity-induced shortened lifespan of the db/db mice. Very recently the efficacy of AdipoRon was studied in experimental periodontitis using diet-induced obese mice (47). In this model, the small molecule AdipoR agonist reduces osteoclast numbers and alveolar bone loss likely though inhibition of osteoclast differentiation.

AdipoRon is shown to elicit adiponectin-like activities in a wide spectrum of animal models. Covering all these reports are beyond the scope of this general review on adiponectin receptor response modifiers. Below is a brief selection focusing on the breadth of the studies. AdipoRon treatment of vascular smooth muscle cells (VSMC) leads to inhibition of platelet-derived growth factor-induced cell proliferation, DNA synthesis, and cyclin D1 expression with an IC_50_ of 25–50 μM (48). In a mouse model of vascular remodeling caused by proliferation of VSMC, oral administration of 50 mg/kg AdipoRon diminishes arterial injury-induced neointima formation by ~57%. Likewise, orally administered in a mouse cardiac remodeling model, AdipoRon alleviates cardiac hypertrophy and fibrosis induced by pressure overload and inhibits angiotensin II-induced TGF-β1 expression as well as cardiac fibroblast differentiation through AMPKα activation (49).

The activity of AdipoRon was extensively studied in mouse models of diseases similar to those investigated for the efficacy of peptidic AdipoR agonists. AdipoRon inhibits pancreatic cancer cell proliferation, colony formation, and anchorage independent growth while increases apoptosis with an IC_50_ of ~25 μM (50). In an orthotopic pancreatic cancer model, AdipoRon at 5 mg/kg/day (administration mode not disclosed) for 14-days reduces tumor size and tumor area per tumor section by 50–75%. Administered intraperitoneally at 1 mg/kg/day for 21-days to mice, AdipoRon reverses corticosterone-induced depression-like state (51). AdipoRon exerts pleiotropic actions on hippocampal neurogenesis, neuroinflammation, serotonergic neurotransmission, and the tryptophan metabolic pathway explaining its antidepressant properties. In mice with acute hepatic injury induced by d-galactosamine, AdipoRon attenuates liver inflammation as read by dwindling proinflammatory macrophage infiltration, shrinkage of pro-inflammatory cytokine production including TNF-α, TGF-β1, IL-1β, and IL-6, meanwhile promoting AMPK activation by phosphorylation (52).

In a mouse model identical to that used for peptidomimetic ADP355, AdipoRon prevents the development of dermal fibrosis. At a concentration of 20 μM, AdipoRon blocks TGF-β-induced fibrotic responses and given orally for 28 days significantly attenuates bleomycin-induced dermal thickness increase while augmenting the dermal white adipose tissue layer (53). When AdipoRon was directly compared with ADP355, low-density lipoprotein receptor expression was more extensive by using 25 nM of the peptidomimetic than 10 μM of the small molecule AdipoR agonist in HepG2 cells (37). A comparison of selected *in vitro* and *in vivo* efficacies of various adiponectin receptor response modifiers is found in Table 2.

**Table 2:** Published activities of adiponectin receptor response modifiers.

**Molecule**	***in vitro* activity approx. IC_**50**_**	***in vivo* efficacy (mg/kg, duration, administration mode)**	**References**
AdipoRon	Activation of LDLR 5 μM	Reversal of corticosterone-induced depression (1 mg/kg in mice, ip, 21 d)	(37) (51)
GTDF	Enhanced of glucose uptake Activity observed at 10 nM	Improvement of metabolic health 10 mg/kg, po, 30 d	(54)
ADP355	Activation of LDLR 25 nM	Complete prevention of protease-inhibitor-induced memory decline (1 mg/kg in mice, ip, 14 d)	(37) (38)
ADP399	Cancer cell proliferation inhibition 10 nM		(33)
Pep70	HSC proliferation inhibition 10 μM		(42)
BHD-1028	AMPK activation <800 nM		(41)
ADP400	900 (EC_50_)		(33)

*LDLR, low density lipoprotein receptor; ip, intraperitoneally; po, orally; HSC, hepatic stellate cells; AMPK, 5′ adenosine monophosphate-activated protein kinase*.

## Additional Non-peptidic Adiponectin Receptor Agonists

Two additional flavonol-type compounds exhibit adiponectin-like activities. GTDF [6-C-β-d-glucopyranosyl-(2S,3S)-(+)-5,7,3′,4′-tetrahydroxydihydroflavonol, Figure 2] is a natural analog of the dietary flavonoid quercetin (54). In a competition radioligand assay using C2C12 myotubes, GTDF interacts with both AdipoRs, with a preference for AdipoR1 at 10 nM that is a magnitude better than the native compound quercetin and dihydroquercetin, the latter being the aglycon of GTDF. The AdipoR1 agonist induces adiponectin-associated signaling and enhances glucose uptake and fatty acid oxidation *in vitro* and improves metabolic health (elevation of glucose clearance, β-cell survival, reduced steatohepatitis, browned white adipose tissue, and improvement of lipid profile) in an AdipoR1-expressing but not an AdipoR1-depleted strain of diabetic mice (54). The dose of GTDF in this instance was 10 mg/kg/day orally for 30 days. Please note the remarkably improved pharmacological parameters of GTDF compared to AdipoRon.

Tiliroside is a glycosidic flavonoid and is found in several dietary plants (55). At a very high dose of 100 mg/kg/day, tiliroside enhances fatty acid oxidation through improved adiponectin signaling and ameliorates obesity-induced metabolic disorders including hyperinsulinemia and hyperlipidemia (56). Nevertheless, tiliroside treatment does not suppress body weight gain and visceral fat accumulation in obese mice. While AdipoR1 and AdipoR2 mRNA expression levels are up-regulated by tiliroside treatment, direct binding to the receptors have not been reported yet.

## Antagonists

Currently only a very limited number of AdipoR antagonists exist although these could find application in diseases characterized by adiponectin overproduction. In this regard, plasma adiponectin levels are reproducibly higher in severe rheumatoid arthritis patients than in the control group (57). Likewise, both AdipoR subtypes are overexpressed in lesional compared to non-lesional areas of osteoarthritis cartilage (58). Conceivably AdipoR antagonists may find applicability as a therapy option in arthritic diseases. Nevertheless, this statement has to be taken with a grain of salt, as tocilizumab (an IL-6 receptor antagonist) treatment of rheumatoid arthritis patients results in both improved clinical findings and increased serum adiponectin levels (59). Another potential initiative for finding adiponectin receptor antagonists is that both AdipoR1 and AdipoR2 are involved in energy metabolism but were reported to have opposing effects suggesting a potential therapeutic utility of negative AdipoR response modifiers in some cardiovascular conditions (60). Indeed, high adiponectin concentrations are associated with increased cardiovascular mortality in patients with congestive heart failure (61, 62), in coronary heart disease and myocardial infarction (63, 64) as well as in chronic kidney disease (65). These studies suggest that once a chronic condition is established, the reverse to traditional adiponectin epidemiology can be observed (66).

However, given the multiple physiological advantages of high levels or circulating adiponectin, the clinical development of negative adiponectin receptor response modifiers without noticeable negative side effects seems extremely difficult at this point. Yet, AdipoR antagonists would represent a much-desired target validation tool for AdipoR agonist drug development.

The first AdipoR antagonist was derived by the agonist peptidomimetic ADP355 by using peptide chemistry tools of the trade proven successful in converting leptin-based ObR agonist peptides into picomolar antagonists (67). Screening of active site library but this time focusing on antagonist properties, a novel octapeptide (ADP400, Table 1) was designed that counteracts ADP355- and its dimer-mediated effects on cancer cell growth at nanomolar concentrations [(33); Table 2]. ADP400 induces mitogenic effects in MCF-7 breast cancer cells perhaps due to antagonizing endogenous adiponectin actions or acting as an inverse agonist.

A ceramidase inhibitor, 1S,2R-D-erythro-2-N-myristoylamino-1-phenyl-1-propanol (MAPP), antagonizes AdipoR signaling at low nanomolar concentrations likely through inverse agonist properties (68). The same yeast-based assay identified TNFα as a potential AdipoR1 antagonist albeit at a 10-fold reduced activity level compared to MAPP. While in biochemistry terms the findings are noteworthy, the fact that agonist is not required to activate the proxy downstream signaling pathway casts shadow to potential significance of the findings in drug development pharmacology.

## Adiponectin Action Mimicking Peptides and Proteins

Biooligomers and biopolymers can either induce adiponectin production in cells and tissues or their synthesis can be triggered by adiponectin in a downstream process. Since these peptides and proteins may serve as adiponectin replacement effectors in various biological processes their potential as therapeutic agents are briefly discussed here.

Upon exposure to adiponectin, B-cells are able to inhibit T-cell trafficking by secreting a 14-mer peptide with a sequence SVTEQGAELSNEER and named PEPITEM (69). PEPITEM is a fragment of the 14.3.3.ζδ protein and binds cadherin-15 on endothelial cells, promoting synthesis and release of sphingosine-1 phosphate ultimately inhibiting T-cell trafficking. In various mouse models of hepatic ischemia-reperfusion injury, *Salmonella typhimurium* infection, peritonitis, uveitis and Sjögren's syndrome, PEPITEM reduces T-cell recruitment into inflamed tissues. From a drug development point of view, the presented animal models employ very high peptide doses (15 mg/kg bolus and 5 mg/kg chronic treatments intraperitoneally or 6 μg peptide directly to the eye). These are significantly higher than those reported for ADP355, an adiponectin-derived anti-inflammatory therapy option (*vide infra*).

Secretion of adiponectin is a highly complex process that is influenced by many factors (70). A long range of agents can increase adiponectin production in cells and tissues, but currently these are far from being therapeutically significant. The following is just as a brief outline. The TNF-α antagonist etanercept increases AMPK phosphorylation in mice, and exerts cardioprotective effects partially due to the upregulation of adiponectin (71). Likewise, safflomide (N-caffeoyltryptamine), a serotonin receptor antagonist, upregulates adiponectin expression in high fat diet-fed rats, resulting in significant reduction in body weight, visceral fat, and improved insulin resistance (72). Improved hepatic insulin sensitivity by the cannabinoid receptor 1 antagonist rimonabant is due to upregulation of adiponectin (73). Rimonabant in high fat-fed dogs enhances hepatic insulin clearance, a process that appears to be linked to an upregulation of the adiponectin pathway.

Activation of PPAR-γ, one of the central regulators of adipocyte biology, improves the secretory profile of the adipose tissue, promoting the release of insulin-sensitizing adipokines, including adiponectin, and reducing inflammatory cytokines (74). It is not surprising that upregulation of adiponectin activity is frequently attempted by enhancing PPAR-γ-mediated biological processes. Rosiglitazone (trade name Avandia) is a small molecule antidiabetic drug and is a PPAR-γ agonist. A recent mode-of-action study revealed that rosiglitazone, similar to other thiazolidinedione insulin sensitizers, elicits an adiponectin-mediated action at the adipose tissue-liver axis in obese rats (75). In this instance, rosiglitazone ameliorates hepatic and systemic insulin resistance, hepatic inflammation, and fatty liver. Rosiglitazone passes the blood-brain barrier-and elicits antidepressant- and anxiolytic-like behavioral effects in mice accompanied by a concurrent increase in serum adiponectin levels (76). Pretreatment with the PPAR-γ-selective antagonist GW9662 blocks rosiglitazone-induced adiponectin production and the central nervous system-resident effects validating PPAR-γ as target of the drug and its positive regulatory role in adiponectin expression. CCK-8 (commercial name Sincalide) is the C-terminal octapeptide fragment of cholecystokinin. CCK-8 stimulates adiponectin production in rat white adipose tissue through a mechanism involving both AKT and PPAR-γ (77). Since CCK-8 actions are only observed in the presence of insulin, it is contemplated to have translational value in novel insulin-sensitizing therapies. Another target validation assay used telmisartan, a dual angiotensin II receptor antagonist and partial PPAR-γ receptor agonist to document a significant reduction of myocardial damage induced by ischemia/reperfusion where the activity is associated with increased adiponectin production and a reduction in certain markers of inflammation (78).

The previous report leads us to additional PPAR subtypes. Fimasartan is a non-peptide angiotensin II receptor antagonist used for the treatment of hypertension and heart failure. In HepG2 cells and liver tissues, fimasartan increases the protein levels of PPAR-δ. It also increases the adiponectin level in visceral fat tissues (79). In target validation experiments, the anti-adipogenic effects can be offset with the PPAR-δ antagonist GSK0660. In differentiated adipocytes the PPAR-β/δ agonist GW501516 induces adipocyte differentiation and the expression of adiponectin in a concentration-dependent manner and ultimately improves insulin sensitivity by increasing the expression of the insulin receptor (80). In an only partially elucidated yet nutritionally interesting aspect, dietary capsaicin may reduce obesity-induced glucose intolerance by suppression of inflammatory responses and enhancement of fatty acid oxidation in adipose tissue and the liver (81). During this process, inflammatory cytokine production decreases and adiponectin in the adipose tissue and PPAR-α in the liver increases.

While some of these studies did experiment with concentration- and dose-response characterization of existing drugs and drug-like adiponectin activity enhancers, their full pharmacological characterization relative to direct adiponectin replacement therapies seems to be available only in a distant future.

## Conclusions and Further Perspective

Based on the dire need for adiponectin receptor response modifiers and the numerous promising candidates in preclinical development it is very likely that adiponectin receptor agonists will enter human clinical trials in the near future. Allysta Pharmaceuticals plans to initiate human trials in late 2019 against dry eye disease using ADP355 as eye drops. From the small molecule agonists listed here, at the time of writing this report, AdipoRon is not subject to any human trial (82). A potential reason for this is that systemic drug formulations have to go through stricter toxicity measures than topical dosage forms.

Drugs targeting adipokine receptors frequently exhibit bell shaped activity curves *in vitro* and a dose-dependent antagonist-agonist switch *in vivo*, leptin variations being prime examples (44). With the numerous proven activities of adiponectin receptor agonists and possibly antagonists we are facing a long process of identifying optimal doses, dose timing and administration modes or formulations that induce only the desired human responses without any unwanted side effects. In addition, coming again from the leptin experience, elevated levels of adipokines may not necessarily mean high levels of active circulating proteins. For example, elevated adiponectin levels may indicate disease progression where adiponectin plays a protective role and is excessively produced. Strong evidence for a possible correlation between adiponectin levels and progressive liver fibrosis in patients with chronic hepatitis B virus infection is observed (83).

In this regard, the generally observable fast renal clearance of peptide drugs (84) may even come handy. Peptides hit their receptors fast, keep them activated/deactivated long but leave the circulation within 1 h. With proper dosing, formulation and perhaps fine-tuning the sequences, peptidic adipokine receptor modifiers can be tailored to induce only desired activities without expanding the pharmacology for an extended period of time and potentially bringing out toxic reactions in patients.

## Author Contributions

LO collected the cited papers and wrote the entire manuscript.

### Conflict of Interest Statement

LO is President of OLPE, LLC, a pharmaceutical consulting firm and co-inventor of the issued US patent on ADP355. LO is a senior consultant of Allysta, Inc., a biotechnology company focusing on the clinical development of ADP355 against various human diseases. The reviewer GS declared a shared affiliation, though no other collaboration, with the author to the handling editor.

## Abbreviations

ACC: acetyl-CoA carboxylase
AdipoR: adiponectin receptor
AdipoRon: orally available small molecule adiponectin receptor agonist
ADP355: first generation adiponectin receptor peptide agonist
ADP399: second generation peptidic adiponectin receptor agonist, linear dimer of ADP355
ADP400: adiponectin receptor antagonist/inverse agonist
AKT: protein kinase B
AMPK: 5′ adenosine monophosphate-activated protein kinase
C/EBP: CCAAT enhancer binding protein
CCK: cholecystokinin
EC: effective concentration
eNOS: endothelial nitric oxide synthase
ERK: extracellular-signal-regulated kinase
FAK: focal adhesion kinase
gAd: adiponectin protein C-terminal globular domain
GTDF: designer quercetin analog
HSC: hepatic stellate cells
IC: inhibitory concentration
IL: interleukin
K_d_: dissociation constant
LDLR: low-density lipoprotein receptor
MAPP: ceramidase inhibitor
PCSK9: proprotein convertase subtilisin/kexin type 9
PPAR: peroxisome proliferator-activated receptor
siRNA: small interfering ribonucleic acid
SMA: α-smooth muscle actin
SMAD: signal transducers for receptors of the transforming growth factor β
STAT3: signal transducer and activator of transcription 3
TGF: transforming growth factor
TIMP: tissue inhibitor of metalloproteinases
TNF: tumor necrosis factor
VSMC: vascular smooth muscle cells.
